# (p)ppGpp: Magic Modulators of Bacterial Physiology and Metabolism

**DOI:** 10.3389/fmicb.2020.02072

**Published:** 2020-09-07

**Authors:** Wieland Steinchen, Victor Zegarra, Gert Bange

**Affiliations:** Department of Chemistry, Center for Synthetic Microbiology (SYNMIKRO), Philipps-University Marburg, Marburg, Germany

**Keywords:** stringent response, (p)ppGpp, alarmones, metabolism, physiology

## Abstract

When bacteria experience growth-limiting environmental conditions, the synthesis of the hyperphosphorylated guanosine derivatives (p)ppGpp is induced by enzymes of the RelA/SpoT homology (RSH)-type protein family. High levels of (p)ppGpp induce a process called “stringent response”, a major cellular reprogramming during which ribosomal RNA (rRNA) and transfer RNA (tRNA) synthesis is downregulated, stress-related genes upregulated, messenger RNA (mRNA) stability and translation altered, and allocation of scarce resources optimized. The (p)ppGpp-mediated stringent response is thus often regarded as an all-or-nothing paradigm induced by stress. Over the past decades, several binding partners of (p)ppGpp have been uncovered displaying dissociation constants from below one micromolar to more than one millimolar and thus coincide with the accepted intracellular concentrations of (p)ppGpp under non-stringent (basal levels) and stringent conditions. This suggests that the ability of (p)ppGpp to modulate target proteins or processes would be better characterized as an unceasing continuum over a concentration range instead of being an abrupt switch of biochemical processes under specific conditions. We analyzed the reported binding affinities of (p)ppGpp targets and depicted a scheme for prioritization of modulation by (p)ppGpp. In this ranking, many enzymes of e.g., nucleotide metabolism are among the first targets to be affected by rising (p)ppGpp while more fundamental processes such as DNA replication are among the last. This preference should be part of (p)ppGpp’s “magic” in the adaptation of microorganisms while still maintaining their potential for outgrowth once a stressful condition is overcome.

## The Metabolism of (p)ppGpp

Bacteria must adapt to fluctuations in their ever-changing surroundings to survive. In order to accomplish optimal resource allocation upon facing environmental shifts, the pathways through which microorganisms rewire their metabolism need to be finely and promptly tuned. An efficient control of metabolism senses stress, adjusts growth accordingly, mandates which genes – and to which extent – are expressed, and ultimately provides a fitness advantage over poorly-adapted microbial competitors. The second messenger molecules (p)ppGpp are compounds that can accomplish all this.

More than 50 years ago, the “magic spots”, which were identified as guanosine 5'-diphosphate 3'-diphosphate (ppGpp) and guanosine 5'-triphosphate 3'-diphosphate (pppGpp) and collectively referred to as (p)ppGpp or “alarmones”, were discovered by Cashel and Gallant (1969). Since then, the synthesis and degradation of (p)ppGpp as well as the (p)ppGpp-mediated response to nutrient limitations, a phenomenon known as the “stringent response”, have been subject of extensive studies. Central in the metabolism of (p)ppGpp are members of the RelA/SpoT homology (RSH)-type protein family (Atkinson et al., 2011). Briefly, when bacteria encounter nutrient-limiting conditions, RSH proteins utilize ATP as a donor substrate and, through transfer of its β‐ and γ-phosphates onto the 3'-hydroxy group of the acceptor substrate guanosine 5'-diphosphate (GDP) or guanosine 5'-triphosphate (GTP), generate ppGpp or pppGpp, respectively (Sy and Lipmann, 1973). RSH proteins also degrade (p)ppGpp through removal of the 3'-pyrophosphate moiety of (p)ppGpp, thereby regenerating GDP/GTP (Hogg et al., 2004). “Long” RSH proteins harbor (p)ppGpp hydrolase and synthetase domains whose reciprocal activities are controlled by further regulatory domains (Atkinson et al., 2011). “Short” RSH proteins consisting only of a (p)ppGpp synthetase or hydrolase domain constitute the class of small alarmone synthetases (SAS) and hydrolases (SAH), respectively (Atkinson et al., 2011). Enzymes of the GppA/PPX family are able to convert pppGpp to ppGpp opening the avenue for more intricate differential regulation by the two alarmone species (Keasling et al., 1993; Kuroda et al., 1997; Kristensen et al., 2008).

The activity of RSH proteins is subject to regulation by various mechanisms, and (p)ppGpp-inducing conditions include, e.g., the presence of stalled ribosomes (Rel/RelA; Haseltine and Block, 1973; Wendrich et al., 2002), fatty acid starvation, and carbon downshifts (SpoT; Battesti and Bouveret, 2006, 2009), enhanced transcription of (p)ppGpp synthetases through cell wall stress stimuli (SAS2/RelP; Zweers et al., 2012; Geiger et al., 2014) or allosteric stimulation through the alarmone pppGpp itself (SAS1/RelQ; Gaca et al., 2015; Steinchen et al., 2015). The intracellular concentrations of (p)ppGpp during growth of *Escherichia coli* amount approximately 10–40 μM during logarithmic growth – unfortunately those basal levels are still not robustly determined because they typically fall beneath or close to the limit of quantification as in two recent studies (Varik et al., 2017; Zbornikova et al., 2019) – and peak at 800 μM at the onset of stationary phase. Full induction of the stringent response during acute amino acid starvation [e.g., induced by a transfer RNA (tRNA) synthetase inhibitor] gives rise to an increase of the intracellular (p)ppGpp concentrations to approximately 1 mM (Haseltine and Block, 1973; Kuroda et al., 1997; Kriel et al., 2012; Varik et al., 2017), ultimately causing growth arrest (Schreiber et al., 1991; Svitil et al., 1993; Potrykus and Cashel, 2008).

## A Priority Program of (p)ppGpp Shutdown Is Advised by Its Binding Affinities

Substantial progress has been made in the identification and characterization of (p)ppGpp binding targets (reviewed in: Kanjee et al., 2012; Steinchen and Bange, 2016; recent original works: Corrigan et al., 2016; Zhang et al., 2018; Wang et al., 2019), which fall into different cellular processes such as DNA replication, transcription, translation, ribosome biogenesis, or nucleotide metabolism (Srivatsan et al., 2008; Liu et al., 2015a; Corrigan et al., 2016; Bennison et al., 2019). Firstly, these studies provide insights as to how (p)ppGpp affect virulence, pathogenicity, persister cell and biofilm formation, heat shock response, and cell growth (Dalebroux and Swanson, 2012; He et al., 2012; Bager et al., 2016; Strugeon et al., 2016; Schafer et al., 2020). Secondly, they supply a wealth of biochemical data that quantitatively describe (p)ppGpp/protein interactions.

**Figure 1:**
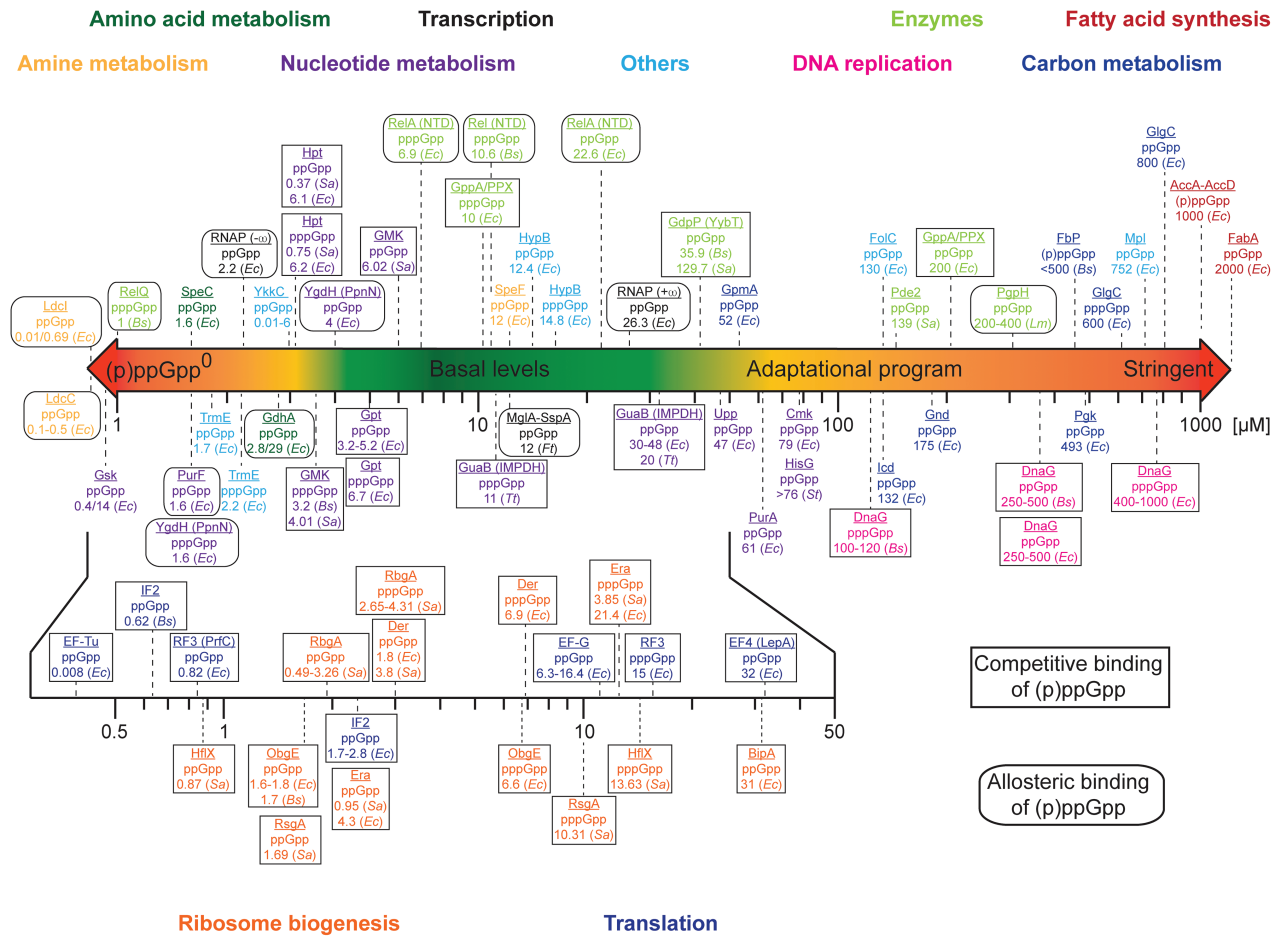
Scheme of prioritization of (p)ppGpp-mediated adaptation. The colored bar denotes the approximate intracellular concentrations (in μM) of (p)ppGpp in bacteria. Binding targets of (p)ppGpp were sorted according to their dissociation constants (K_d_). For DnaG, “enzymes” and proteins of carbon metabolism and fatty acid synthesis, K_i_ or EC_50_/IC_50_ values are depicted. Proteins are color-coded according to their belonging to different groups/biochemical processes. Rectangles and rounded rectangles indicate whether (p)ppGpp binding to the target molecule is competitive or allosteric. Bacterial species are abbreviated as follows: *Escherichia coli* (*Ec*), *Bacillus subtilis* (*Bs*), *Staphylococcus aureus* (*Sa*), *Listeria monocytogenes* (*Lm*), *Francisella tularensis* (*Ft*), *Thermus thermophilus* (*Tt*), and *Salmonella typhimurium* (*St*). Further details can be found in Supplementary Table S1.

We were wondering whether any prioritization in the order of regulation between those targets/processes would exist. We collected and analyzed binding affinities (exemplified by the dissociation constants, K_d_, of the (p)ppGpp/protein complexes), inhibitory constants (K_i_) and EC_50_/IC_50_ values for the targets of (p)ppGpp and depicted a scheme of prioritization in (p)ppGpp modulation (Figure 1 and Supplementary Table S1). Proteins involved in the metabolism of amines and amino acids exhibit very high-affine (p)ppGpp binding with K_d_’s as low as 0.01 μM (*E. coli* LdcI, Kanjee et al., 2011a,b). They are followed by a multitude of enzymes involved in the metabolism of nucleotides featuring K_d_ values below 10 μM (Zhang et al., 2018; Wang et al., 2019), the tRNA modification GTPase TrmE and riboswitches of the YkkC type 2a (Peselis and Serganov, 2018; Sherlock et al., 2018). Notably, their K_d_ values below the assumed basal levels of (p)ppGpp imply a certain degree of regulation by (p)ppGpp even under non-stringent conditions (see below). (p)ppGpp binding to targets involved in transcription [i.e., *E. coli* RNA polymerase (RNAP); Bhardwaj et al., 2018] and *Francisella tularensis* MglA-SspA (Cuthbert et al., 2017) proceeds between 2 and 25 μM. It shall be noted that (p)ppGpp elicit control over *E. coli* RNAP through modulation of relative transcription rates from different promoters instead of a general reduction or induction of RNAP activity (Gourse et al., 2018). These targets are succeeded by further enzymes of nucleotide metabolism with K_d_ values ranging between 30 and 80 μM. Except for GpmA exhibiting a K_d_ of 52 μM, proteins associated with carbon metabolism feature dissociation constants/IC_50_ values of 132–800 μM (Dietzler and Leckie, 1977; Taguchi et al., 1977; Fujita and Freese, 1979; Wang et al., 2019). In a similar range falls the inhibition of DNA replication *via* (p)ppGpp binding to DnaG (Wang et al., 2007; Maciag et al., 2010; Rymer et al., 2012). Enzymes of fatty acid metabolism exhibit weak binding affinities (Polakis et al., 1973; Stein and Bloch, 1976), suggesting regulation by (p)ppGpp only to take place under full stringent control (i.e., 1,000 μM (p)ppGpp). Some enzymes partaking in (p)ppGpp metabolism, i.e., the (p)ppGpp synthetases RelQ (Gaca et al., 2015; Steinchen et al., 2015) and Rel/RelA (Shyp et al., 2012; Kudrin et al., 2018; Takada et al., 2020a), are stimulated in their activity by pppGpp and to a lesser extent by ppGpp. Furthermore, the enzymes GdpP, Pde2, and PgpH involved in the degradation pathway of the second messenger cyclic-diadenosine-monophosphate (c-di-AMP) display K_i_ values for inhibition by ppGpp between 35 and 400 μM.

Importantly, in proteins where (p)ppGpp bind competitively to another compound (Figure 1 and Supplementary Table S1), the intracellular concentration of this compound also affects the fraction of the (p)ppGpp-bound protein. The influence of this other compound rises with its intracellular concentration. For example, the concentration of GTP under non-stringent conditions is assumed to fall between approximately 1 (Varik et al., 2017; Zbornikova et al., 2019) and 5 mM (Bennett et al., 2009) but may drop heavily due to (p)ppGpp-dependent inhibition of GTP anabolism (Kriel et al., 2012; Liu et al., 2015b). Thereby, inhibition of GTPases is governed by the (p)ppGpp to GTP ratio that is indicative for the metabolic state of the cell. Assuming, for example, similar K_d_ values for (p)ppGpp and GTP binding, only approximately 1% of a protein would be inhibited at 10 μM (p)ppGpp/1 mM GTP while this fraction rises to 50% at equal concentrations of both nucleotides. In fact, the K_i_ for the inhibition of the GTPase RbgA of 300 and 800 μM (ppGpp and pppGpp in presence of 50S ribosomal subunits determined at 1 mM GTP; Pausch et al., 2018; Supplementary Table S1) is a better estimate than the K_d_ value. Similar considerations apply to some enzymes of nucleotide metabolism, e.g., GMK (Nomura et al., 2014) with its substrate GMP or Gpt (Hochstadt-Ozer and Cashel, 1972) with the substrate guanine. In contrast to GTP, however, the intracellular concentrations of those nucleotides are lower, i.e., 24 μM GMP and 19 μM guanine (Bennett et al., 2009), suggesting 90% inhibition at (p)ppGpp concentrations of approximately 200 μM. In contrast, the enzymes YgdH (PpnN; Zhang et al., 2019), PurF (Wang et al., 2019), and LdcI are allosterically inhibited by (p)ppGpp, rendering inhibition by (p)ppGpp independent of the substrate concentration. Summarized, the kinetic parameters of (p)ppGpp interaction with target proteins indicate the following hierarchy of adaptation: amine and amino acid metabolism, nucleotide metabolism, translational and ribosome biogenesis GTPases, DNA replication, carbon metabolism, and fatty acid synthesis (Figure 1).

## Importance of Basal (p)ppGpp Levels for Bacterial Physiology

Increasing evidence recommends that bacteria require basal (p)ppGpp levels to maintain homeostasis (Sokawa et al., 1975; Sarubbi et al., 1988; Silva and Benitez, 2006; Lemos et al., 2008; Gaca et al., 2013; Stott et al., 2015; Li et al., 2020) and strains that are completely devoid of any (p)ppGpp (known as (p)ppGpp^0^) reveal multiple disabilities in cell division, transcription, translation, GTP homeostasis, and antibiotic tolerance. In *Bacillus subtilis*, the lack of (p)ppGpp causes elevated GTP levels, which through dysregulation of the GTP-dependent transcriptional repressor CodY result in auxotrophies for branched-chain amino acids (Kriel et al., 2014). Similar CodY-mediated detrimental effects of ppGpp^0^-strains were observed for *Enterococcus faecalis* (Gaca et al., 2013) and *Staphylococcus aureus* (Pohl et al., 2009). In a *rel*-deletion strain of *Synechococcus elongatus*, the transcript levels of 52–67% of all genes were upregulated at least 3-fold and the levels of rRNA elevated indicating a “transcriptionally relaxed” state (Puszynska and O’Shea, 2017). Furthermore, cell size and volume increased but could be restored by synthetic compensation of (p)ppGpp (Puszynska and O’Shea, 2017). In *Vibrio cholera* basal (p)ppGpp has been linked to the expression of virulence factors and cell motility (Silva and Benitez, 2006). Basal (p)ppGpp was also required for the tolerance of *E. faecalis* against vancomycin (Abranches et al., 2009). These reports emphasize the importance of basal (p)ppGpp levels, which through high-affine binding of the alarmones (K_d_ < 10 μM; Figure 1) exert a regulatory function in the absence of a (p)ppGpp-stimulating trigger.

## Toxic Over-Accumulation of (p)ppGpp

Intuitively, a prolonged mismatch of (p)ppGpp synthesis and degradation resulting in over-accumulation of (p)ppGpp, is detrimental for the cell. In *E. coli*, RelA and SpoT are responsible for the synthesis of (p)ppGpp, with only SpoT being able to hydrolyze the alarmones. As such, the role of SpoT is pivotal for preventing toxic (p)ppGpp accumulation as exemplified by SpoT-deletion strains being characterized by reduced growth rates and distorted amino acid requirements (Xiao et al., 1991). Bacteria of the Firmicutes phylum, instead of possessing RelA and SpoT, harbor the bifunctional enzyme Rel (Atkinson et al., 2011). The hydrolase activity of Rel is required to prevent toxic concentrations of (p)ppGpp, which results in severe growth defects (Gratani et al., 2018; Takada et al., 2020b). This effect appears more notorious in the presence of the two small alarmone synthetases, RelP and RelQ, the (p)ppGpp synthetase activity of which is not properly contained in absence of the (p)ppGpp-degrading Rel (Geiger et al., 2014). In *Mycobacterium tuberculosis*, (p)ppGpp degradation is equally essential since deletion of (p)ppGpp hydrolase activity, while retaining (p)ppGpp synthesis, lead to lethal toxicity evidenced by irregularities in colony formation irrespective of the nutritional environment (Weiss and Stallings, 2013). Notably, these *M. tuberculosis* strains were also impaired in their virulence in a mouse model during both acute and chronic infection, highlighting the interference with (p)ppGpp metabolism as a potential antimicrobial therapeutic strategy. Taken together, a tight regulation of the antagonistic (p)ppGpp producing and degrading activities is indispensable for many, if not all bacteria for (i) correctly adjusting (p)ppGpp levels in response to environmental cues, (ii) coordinating various cellular processes in an orchestrated manner, and (iii) avoiding lethal consequences due to (p)ppGpp over-accumulation.

## Crosstalk Between (p)ppGpp and c-di-AMP

Bacteria possess a thorough toolbox of nucleotide-based second messengers to efficiently respond to diverse external cues (Pesavento and Hengge, 2009; Kalia et al., 2013; Hengge et al., 2016). Among those, c-di-AMP is a signaling molecule mainly synthetized by Gram-positive bacteria (Romling, 2008; da Aline Dias et al., 2020; He et al., 2020; Yin et al., 2020), which fulfills a pivotal role in the osmotic homeostasis (Stulke and Kruger, 2020). The dynamic range of intracellular c-di-AMP is much lower than for (p)ppGpp and lies in the one-digit μM range (in *B. subtilis* 1.7 μM during vegetative growth to 5.1 μM 2 h after sporulation; Oppenheimer-Shaanan et al., 2011). The importance of c-di-AMP for bacterial physiology is also highlighted by the observation that both the complete absence and over-accumulation of c-di-AMP impede growth of *B. subtilis* (Mehne et al., 2013; Gundlach et al., 2015). Comparison of the transcriptional profiles of *S. aureus* cells at high c-di-AMP or (p)ppGpp concentrations revealed an overlap of 27.9% between the two regulons (Corrigan et al., 2015). Intriguingly, (p)ppGpp inhibit the activity of the *S. aureus* c-di-AMP phosphodiesterase GdpP with a K_i_ of 129.7 ± 42.8 μM *in vitro* correlating with higher levels of c-di-AMP observed *in vivo* (Corrigan et al., 2015). Inhibition by ppGpp with an IC_50_ of 139 ± 5.6 μM was also reported for the second *S. aureus* c-di-AMP phosphodiesterase Pde2 (Bowman et al., 2016). Similar observations made for the c-di-AMP hydrolases YybT (GdpP) from *B. subtilis* (Rao et al., 2010) and PgpH from *Listeria monocytogenes* (Huynh et al., 2015). These K_i_/IC_50_ values suggest that (p)ppGpp concentrations must rise above their basal levels to inhibit c-di-AMP degradation. Deletion of *S. aureus* GdpP with concomitant increase of c-di-AMP evokes elevated (p)ppGpp (Corrigan et al., 2015), thus implying that induction of one second messenger, (p)ppGpp or c-di-AMP, augments the other. Conversely, the depletion of the only c-di-AMP synthetase, *dacA*, in *L. monocytogenes* also induced a toxic accumulation of (p)ppGpp (Whiteley et al., 2015). This apparent twist might be caused in the narrow dynamic range of c-di-AMP whereby deflection in either direction triggers (p)ppGpp synthesis or indicate species-specific differences in the regulatory circuits. Nevertheless, the functional connection of c-di-AMP and (p)ppGpp is further substantiated by the observation that the essentiality of *dacA* in *L. monocytogenes* during growth in rich medium was abrogated in a (p)ppGpp^0^-strain or in the wild-type strain grown in minimal medium (Whiteley et al., 2015). Hereby, the activity of the GTP-dependent transcriptional regulator CodY appears critical to *dacA* essentiality in rich media in that mutation of *codY* in the (p)ppGpp^0^-strain renders *dacA* again essential. This implies that elements of the CodY regulon may be toxic in the absence of c-di-AMP-producing DacA (Whiteley et al., 2015). The GTP-loading state and activity of CodY (Sonenshein, 2007) are in turn linked to (p)ppGpp through the inhibition of GTP anabolic enzymes involved in nucleotide metabolism by the alarmones (see above). Thus, as a whole, the interdependencies between c-di-AMP and (p)ppGpp conceivably require a high degree of coordination and involve a number of different cellular processes.

## The Emerging Hyperphosphorylated Nucleotides (p)ppApp

In the 1970s, the accumulation of two further magic spots designated as (p)ppApp was observed in *B. subtilis* during the early stages of sporulation (Rhaese and Groscurth, 1976; Rhaese et al., 1977; Nishino et al., 1979) and the (p)ppApp synthetic activity retrieved from the ribosomal fraction, which indicates the contribution of an RSH. The first RSH to catalyze (p)ppApp formation was recently identified in *Methylobacterium extorquens* (Sobala et al., 2019), however, (p)ppApp-producing activity was not evidenced for the *E. coli* RelA enzyme (Jimmy et al., 2020). Another source of (p)ppApp is the nucleoside 5'-diphosphate kinase from *Streptomyces morookaensis* although the enzyme being secreted raises the question of its physiological relevance for this organism (Oki et al., 1975). The high similarity of (p)ppGpp and (p)ppApp – they only differ in their nucleobase – advises a putative overlap of their target spectra. Recent studies evidence that *E. coli* PurF and RNAP, both of which are validated targets of (p)ppGpp, also bind (p)ppApp (Bruhn-Olszewska et al., 2018; Ahmad et al., 2019). However, while in PurF both second messengers bind in similar fashion at the same site and inhibit the enzyme with equal potency (Ahmad et al., 2019; Wang et al., 2019), the binding sites on RNAP are discrete and (p)ppApp enhance transcription from, e.g., the *rrnB* P1 promoter as opposed to (p)ppGpp (Bruhn-Olszewska et al., 2018). In one study, ppApp also inhibited the (p)ppGpp synthetic activity of *E. coli* RelA with an IC_50_ of 24.5 ± 3.5 μM (Beljantseva et al., 2017) raising the possibility that, in fact, (p)ppApp and (p)ppGpp are antagonists. Besides potentially fulfilling regulatory functions in their host cell, (p)ppApp also serve as toxins during interbacterial warfare (Ahmad et al., 2019). Hereby, the type VI secretion system (T6SS) effector protein Tas1 of *Pseudomonas aeruginosa* PA14, a (p)ppApp synthetase, is injected into the target cell and depletes the cellular ATP pools, resulting in dysregulation of the metabolome and, ultimately, cell death. Endogenous ppApp, the metabolism of which is embedded in toxin-antitoxin systems, might represent an intricate pathway to control growth (Jimmy et al., 2020). Whether the (p)ppApp molecules exert growth control independent from (p)ppGpp *via* a separated protein target spectrum or in a competitive manner to (p)ppGpp remains to be investigated.

## Concluding Remarks

The functions of (p)ppGpp are often simplistically portrayed as a biphasic switch between relaxed and stringent environmental conditions, the latter triggering a major metabolic rearrangement through induction of (p)ppGpp synthesis. The presence of gradation in (p)ppGpp-dependent regulation of transcription is an approved mechanism (Traxler et al., 2008; Balsalobre, 2011; Traxler et al., 2011). However, the wide range of binding affinities among the (p)ppGpp-affected protein targets covering four orders of magnitude furthermore suggests a post-translational adaptational program activated hierarchically by increasing (p)ppGpp. The depicted scheme illustrates the importance of high-affine (p)ppGpp targets that explicitly require regulation, thus suggesting a protagonism of the alarmones on cell homeostasis. We believe that the post-translational sequential “dimming” of protein activities by (p)ppGpp, a conception potentially also true for related second messengers like (p)ppApp and c-di-AMP, plays a major role in successful adaptation of microorganisms.

## Data Availability Statement

The original contributions presented in the study are included in the article/Supplementary Material, further inquiries can be directed to the corresponding authors.

## Author Contributions

WS and GB conceived the study. WS and VZ performed the analysis. WS, VZ, and GB wrote the manuscript. All authors contributed to the article and approved the submitted version.

### Conflict of Interest

The authors declare that the research was conducted in the absence of any commercial or financial relationships that could be construed as a potential conflict of interest.
